# Commentary: Stabilizing Constructs through Collaboration across Different Research Fields as a Way to Foster the Integrative Approach of the Research Domain Criteria (RDoC) Project

**DOI:** 10.3389/fnhum.2016.00363

**Published:** 2016-07-19

**Authors:** Walter Glannon

**Affiliations:** Department of Philosophy/Arts, University of CalgaryCalgary, AB, Canada

**Keywords:** brain-mind, environment, mechanistic explanations, psychiatric disorders, psycho-biological interaction, Research Domain Criteria (RDoC)

The Research Domain Criteria (RDoC) project was initiated by the NIMH as a way of explaining and understanding the etiology and pathophysiology of psychiatric disorders at a brain-systems level (Insel et al., 2010; Casey et al., 2013, 2014). By identifying dysfunctional neural circuits and networks, the RDoC aims to provide a framework promoting more accurate diagnosis and prognosis and specify targets that could predict responses to therapeutic interventions for these disorders. This is an improvement over the Diagnostic and Statistical Manual of Mental Disorders (DSM-5), which focuses on symptoms rather than causes.

Jacqueline Sullivan argues that there is a lack of explanatory and conceptual integration in the RDoC (Sullivan, 2016). Its success as a theoretical model for explaining neuropsychiatric disorders depends on collectively stabilizing valid constructs across different research fields in the mind-brain sciences. One feature of this stabilizing strategy is connecting functional analysis with mechanistic explanations (Craver, 2007; Bechtel, 2008), which “identify the physical parts (e.g., systems, cells, molecules) and processes (e.g., activation, firing, phosphorylation) that realize organism-level functions.” Presumably, explanations in neuroscience are mechanistic. Yet by focusing only on neural parts and processes, these explanations provide at best an incomplete account of the development of psychiatric disorders and the experience of the people who have them. They are too limited to explain the extent to which biological, psychological, and environmental factors influence neural function and dysfunction and the role of these factors in diagnosis, prognosis and treatment of these disorders. Sullivan acknowledges criticism of the RDoC as “braincentric” and “decontextualizing mental disorders from their bodily, social, and environmental contexts” (Whooley, 2014; Bernard and Mittal, 2015). Although the integrative aspect of mechanistic explanations may strengthen the RDoC as a theoretical construct, they cannot fully explain the onset and severity of conditions such as depression, anxiety, schizophrenia, or variable patient responses to therapeutic interventions for them. Mechanistic explanations from cognitive psychology and cognitive neuroscience fall short of providing a satisfactory psycho-biological framework in which to gain a better understanding of healthy and diseased brains. This is more likely to come from observing and treating actual subjects and patients in psychiatric research and clinical psychiatry.

Mechanistic explanations emphasize bottom-up causation in which lower-level physical parts and processes realize higher-level physical functions. They fail to capture the bi-directional bottom-up and top-down causal relations between psychological and neural properties in brain-mind and mind-brain interaction. They also fail to capture how other bodily systems and a person's response to the environment can influence the activity of neural circuits and networks. For example, acute or chronic psychosocial stress can cause dysregulation in the hypothalamic-pituitary-adrenal (HPA) axis (Hohne et al., 2014). This can induce sleep, cognitive, and mood disturbances and other symptoms associated with major depression and generalized anxiety. Such dysregulation can result in an adverse biochemical environment in the brain with high circulating levels of norepinephrine released from the adrenal medulla and cortisol released from the adrenal cortex. A prolonged period of excess cortisol may subsequently result in neuronal and synaptic degeneration in the prefrontal cortex and further deleterious effects on cognition, mood, and motivation. Psychosocial stress may also cause hyper-activation of the amygdala and dysregulated fear processing associated with anxiety and panic disorders (LeDoux, 2003). Stressors in the womb can adversely affect fetal brain development and eventually result in neurodevelopmental disorders. On the other hand, cognitive-behavioral therapy may enable some persons with depression to modulate prefrontal cortical function in ameliorating symptoms (Goldapple et al., 2004). This is an example of salutary psycho-biological interaction and top-down neuromodulating effects of psychological states in rewiring brain circuits. In one form of neuro-immune interaction, viral infections can induce neural endothelial and epithelial cells to release proinflammatory cytokines and cause abnormal metabolism of serotonin, dopamine, and norepinephrine in the brain. These cytokines can be psychoactive and induce abnormal emotional states and behavioral changes (Miller et al., 2009). All of these examples illustrate that endocrine, immune, and environmental systems outside the brain can interact with and alter systems inside the brain. These phenomena cannot be explained in terms of parts and processes in the brain alone.

Nor do mechanistic explanations adequately account for the epigenetics of depression. They do not consider how environmental exposures can alter chromatin structure in gene expression in determining the lifetime risk of depression or its severity in those who develop it (Nestler, 2014). Dynamic patterns of gene regulation in response to internal cues such as hormones, neurotransmitters, or cellular states, and external cues in the form of environmental stress, can influence genetic factors regulating neural activity. Explanations focusing on physical parts and processes in the brain fail to consider the influence of these external cues on neural function and dysfunction.

The success of the RDoC in psychiatry will not depend on the theoretical issue of whether it allows for the collective stabilization of valid constructs for conceptual and explanatory integration. Specifically, it will not depend on mechanistic explanations. Rather, it will depend on the combined theoretical and practical issue of whether investigation of neural circuits and networks results in more accurate diagnosis, prognosis and more effective therapies for, and ideally prevention of, diseases of the brain-mind. This would enable the RDoC to achieve or at least approximate the goal of reducing the global burden of these diseases and improving quality of life for the millions of people who suffer from them. Sullivan's proposed stabilizing project may improve the RDoC as a conceptual model for cognitive neuroscience. Yet while the RDoC considers environmental and other factors outside the brain among its domains and is not strictly reductive, it does not adequately appreciate the degree to which psychosocial factors in particular contribute to psychiatric disorders. It is an incomplete model for explaining them. This requires a more comprehensive model that includes the full extent of interaction between and among the central nervous and other bodily systems, and especially interaction between the person constituted by these systems and the environment. To reduce the magnitude of harm from mental illness, psychiatrists need to focus not just on neural parts and the integrated neural functions in which they are realized but also on the whole neurobiological-psychological-environmental context in which people are affected by it.

## Author contributions

The author confirms being the sole contributor of this work and approved it for publication.

### Conflict of interest statement

The author declares that the research was conducted in the absence of any commercial or financial relationships that could be construed as a potential conflict of interest.
